# Is Time Spent Outside the Family Home a Risk Factor for Lead Exposure in Pre-School Children Living in Broken Hill?

**DOI:** 10.3390/ijerph18157721

**Published:** 2021-07-21

**Authors:** David M. Lyle, Frances T. Boreland, Najeebullah Soomro, Melinda Glisson-Gladman

**Affiliations:** 1 Broken Hill University Department of Rural Health, Sydney Medical School, Faculty of Medicine and Health, The University of Sydney, Broken Hill, NSW 2880, Australia; naj.soomro@sydney.edu.au (N.S.); melinda.gladman@bigpond.com (M.G.-G.); 2 Far West Local Health District, Broken Hill, NSW 2880, Australia; 3 Broken Hill Environmental Lead Program, Environmental Protection Agency (EPA), Broken Hill, NSW 2880, Australia; Frances.Boreland@epa.nsw.gov.au

**Keywords:** child and adolescent health, lead and health, rural health, program evaluation

## Abstract

Broken Hill is amongst a group of communities internationally that are at greater risk from lead due to active or historical lead industries. Current evidence suggests there is no safe level of lead for young children. This paper describes places outside the family home where young Broken Hill children spend time and considers the potential for this to contribute to lead risk. We interviewed 65 families of children 3 years old or younger and detailed the top five places children spent time at outside the family home. Exposure to private residences outside the family home was recorded for most (88%) young children. Nearly two thirds stayed there five or more hours per week. Most children went there on a weekly basis over many months (median, 12 months), increasing the likelihood of exposure to lead hazards. Further investigation of the lead hazard and risk behaviour of children at these residences would assist in developing guidelines for remediation of the lead hazard for all private residences in Broken Hill. This approach to elucidating the potential sources and pathways of lead and other heavy metal exposures for young children may have merit in other settings where comprehensive zonal remediation is not feasible or may not be warranted.

## 1. Introduction

Broken Hill, NSW, Australia, is amongst a group of communities internationally that are at greater risk from lead due to the presence of active or legacy lead industries or having a large number of houses with lead-based paint in poor condition [1,2,3,4]. Mining of lead and other base metals has occurred in the area for more than 130 years, and lead was smelted on site for the first fifteen years [5]. The town grew around the mines and the associated waste dumps, and homes were built within walking distance. Some homes are less than 100 m away from mine waste dumps, and all the residential area is within 2.8 km of the mines. Almost all housing within a kilometre of the mines was built before 1940, generally of corrugated iron (roofing iron) cladding over a timber frame; these homes are very permeable to dust ingress unless maintained well. Eighty percent of the homes within the community were built before 1970 and are likely to have lead-based paint. Lead levels in soil and dust are highest closest to the mines, with soils exceeding the current Australian Health Investigation Level of 300 mg/kg for most residential blocks across the town and 3000 mg/kg in the areas close to the mines [6,7,8,9,10,11,12,13]. These levels are substantially higher than those found in urban areas not located close to lead industries [14]. Recent modelling has shown lead levels in dust and soil both significantly impact children’s blood lead level [12,14]. The Broken Hill climate is semi-arid (annual average rainfall, 247.7 mm) [15], and house yards tend to have large areas of bare soil, which adds to the potential for contaminated soil to be resuspended and expose children to lead.

The detrimental health effects of lead in young children have been extensively investigated. Lead impacts many body systems including the central nervous system, kidneys, blood chemistry and reproductive organs, with the greatest concern being the impact on children’s IQ [16,17,18,19,20]. Cognitive impairment associated with exposure to lead during childhood persists well into adulthood [21]. Accidental ingestion during normal hand-to-mouth behaviour and play in contaminated environments is the main way in which children are exposed to lead in Broken Hill [22]. Recent evidence suggests that there is no safe level of lead exposure and that adverse health effects can occur even at very low blood lead levels (<5 µg/dL (BLL)) [23]. In response, there has been an increasing focus on preventing rather than mitigating childhood lead exposure [23,24,25,26]. Community-wide strategies to control lead in soil, paint and dust are effective in reducing lead exposure at the population level [27,28]. However, some communities that have active approaches to managing lead exposure have had difficulty in reducing the BLL to <5 µg/dL [29,30]. For example, since 2010, annual BLL surveys of 1–4-year-old children in Broken Hill have found 41–53% thereof have the BLL of ≥5 µg/dL, 5–9%—≥15 µg/dL.

Since the early 1990s, there have been multiagency efforts to reduce lead exposure in Broken Hill, including a comprehensive blood lead screening program, parental education and community awareness programs, environmental assessment (homes, dust, soil), home remediation, zonal remediation of high lead hazard areas and improved control of emissions from mining. These efforts contributed to a two-thirds reduction in the children’s BLL (from 16.7 µg/dL in 1991 to 5–6 µg/dL since 2015) [22]. Despite this, more than half (52%) of the young children in Broken Hill still have the BLL of ≥5 µg/dL—the current Australian Health guideline for public health notification and risk management of lead exposure [1,31].

A recent Broken Hill study highlighted the significance of early exposure to lead and the widespread distribution of children with a very high BLL (≥10 µg/dL) throughout Broken Hill (with clustering in high lead hazard zones), indicating the need for greater investment in hazard reduction across the whole community [32]. Presently, home risk assessment is offered to families of 12-month-old children irrespective of the BLL and to families of 1–4-year-old children with the BLL ≥ 5 µg/dL and may recommend remediation of lead hazards in the family home. This approach does not extend to systematic examination of other places where children spend time. Home remediation alone has not been sufficient in reducing the children’s BLL in the long term [6] which suggests that other places outside the family home pose a lead risk. These additional places could be targeted for environmental risk assessment and potential remediation in addition to targeted zonal remediation of high lead hazard zones to support early intervention/abatement strategies across the whole community that aim to keep the BLL below 5 µg/dL for individual children.

This study aimed to investigate places outside the family home where young Broken Hill children spend time as a potential source of lead exposure. Based on these findings, we considered the potential value of extending environmental risk assessment and remediation in Broken Hill to include all the places young children may be exposed to lead.

## 2. Materials and Methods

We conducted a descriptive cross-sectional study. Families or carers of children attending the Child and Family Health Lead Screening Program for routine blood testing between February and May 2018 were invited to participate in the study if they resided in Broken Hill and the child was 3 years old or younger. Sixty-five families were interviewed using a structured questionnaire (participation rate, 59%). The sample size gave a margin of error of ±12% for the sample proportion of 50%.

Data collection included the child’s age, blood lead level at the test, family home address, the amount of time spent away from home each week, details of the top five places the child spent time at over the past month (address and type of place, relationship to the family, frequency of visits, duration of visits, other young children present). Parents or carers were asked to nominate locations their child spent time at outside the family home in the previous month. Where the exact number of hours and visits could not be determined, a range (minimum and maximum) was collected for the given location and the minimum time was used for analysis. Locations were ranked in the order of time spent there. The primary location was the one ranked highest. The types of location were categorised as follows: private residence (relative/non-relative), care facility (preschool/family day care), recreational space (outdoor public space/built facilities), commercial, other. When more than one private residence was visited, the main residence was the most commonly visited home, based on the time spent there. The family home and private residence addresses were grouped into five risk areas (1—highest, 5—lowest) based on lead levels in soil, dust and blood as described in previous papers [6]. Ages were grouped as infants (6–11 months) and 1-, 2- and 3-year-olds, which aligned with attendance at one of the scheduled screening points at 6, 12, 18, 24 and 36 months.

The time spent at a private residence each week was used as a proxy measure of lead risk in this study because of its potential value to guide primary prevention in the at-risk population. There are no published data on lead risk associated with the time spent at a residential address (either on a weekly basis or as a cumulative measure over time) so cut points at 5 h and 10 h per week were selected to provide two estimates of the number of children who may have had sufficient time to interact with lead hazards at that site.

We used SPSS (version 24; IBM, Chicago, IL, USA) to analyse the data. One-sample 95% confidence intervals of the sample proportion were calculated using the normal approximation method for key descriptor measures, such as the proportion of children spending ≥5 h per week at a private residence outside the family home. Ethical approval for the project was obtained from the Greater Western Human Research Ethics Committee (LNR/17/GWAHS/113).

## 3. Results

Forty-six (71%) of the children were one- or two-years-old, girls outnumbered boys 6 to 4, and for most participants (82%), the family home was outside the highest soil lead hazard zones (Table 1). All the children spent time away from their own home. Private residences were the most common (primary) place visited (44 children—68%), followed by childcare facilities (10 children—15%). The median time spent at the primary place ranged from 7.5 h per week for infants to 15 h for 3-year-olds (Table 1). Two-thirds of the children went to that place at least weekly in the previous month (89% for private residences) and the parents reported that their child had been going there for 2–36 months (median, 12 months) (Table 2).

Most families reported taking their child to at least three places outside the family home during the previous month (Table 2). The median time spent at the primary place was 14 h, decreasing to 3 and 2 h for the second- and third-ranked places, respectively.

When all the places visited by a child in the previous month were included, 57 children (88%; 95% CI: 77–95) spent time at a private residence away from their own home: 29—at one residence only, 17—at two homes, 11—at 3–5 different homes.

**Figure 1:**
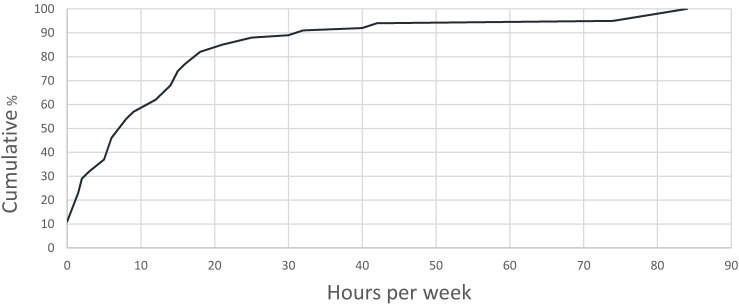
Cumulative frequency (%) graph of the time spent at the private residence (outside the family home) most commonly visited by young children in the last month, Broken Hill, 2018.

The time spent at the private residence most commonly visited by children outside the family home is presented in Figure 1. Eight children (12%) did not make a visit during the previous month, 41 children (63%; 95% CI: 50–75) spent ≥ 5 h per week there, and 28 children (43%; 95% CI: 30–56) spent ≥ 10 h per week. Thirteen (23%) of these most commonly visited homes were in the areas of known high risk of lead exposure located closest to the mining area, and 39 (68%; 95% CI: 56–80) of the home occupants were grandparents (Table 3). Grandparents accounted for 66% of occupants when all the households visited by children were included (99 places).

## 4. Discussion

Primary prevention of lead exposure for young children in high-risk environments such as Broken Hill can be difficult to achieve. It requires a combination of evidence-informed actions that make the environment lead-safe and the use of local data on the potential sources and pathways for lead exposure to guide action. This study provides important information about where young children spend time when away from the family home and shows how this may contribute to their lead risk. Lead is widely dispersed throughout Broken Hill in soil, ceiling dust and paint. Children can be exposed to any of these sources whether they spend time in the family home or elsewhere in Broken Hill, including other private residences, childcare facilities and recreational spaces such as parks and playgrounds.

Time spent at private residences outside the family home is commonplace for young children in Broken Hill. Many spent a substantial amount of time there, with nearly two-thirds of all the children staying five or more hours per week at least at one place. Most children went to these places on a weekly basis over many months (median, 12 months). How typical this is for young children elsewhere is difficult to determine because of the lack of data from other communities and settings. Research in this area tends to report on how children spend their time on different activities rather than where they spend time [33,34,35].

Because lead is so widespread in Broken Hill, these additional residences are likely to pose a lead risk to young children and therefore should be identified for environmental assessment and remediation in addition to the family home where zonal remediation is not planned for logistical or funding reasons. However, we were not able to estimate the magnitude of this risk because it was beyond the scope of this study to collect information on the lead hazard (via environmental assessment) and assess the risk behaviour of children at these locations. Other places where children spent time at, such as childcare facilities and recreational spaces, are potential sources of exposure and should be incorporated in the lead management strategy to mitigate the risk to children at those locations as well.

The study also reflects the multigenerational nature of the Broken Hill community and the significant involvement of grandparents with their grandchildren. Grandparents are an important group in the community to engage with and gain their support for home assessment and remediation that might include their place of residence.

These findings add to the results of an associated longitudinal study [32] that documented lead risk in children across the Broken Hill community. In that study, geospatial mapping identified specific localities, both within the very high soil lead hazard zones and elsewhere, where clusters of children with a very high BLL resided. It also highlighted the significance of early exposure to lead and the need for a primary prevention focus during the first 12 months of life to keep the BLL <5 µg/dL.

At the local level, the goal of keeping the BBL in children below the current national reference level has been difficult to achieve and will require a combination of actions that target both individual and community-wide risk. These includes (i) minimising emissions and the addition of new lead to the environment, (ii) cleaning up the existing ‘legacy’ lead (zonal remediation) in high-risk zones and (iii) a proactive approach to preventing the BLL reaching 5 µg/dL in individual children. The development of an environmental risk assessment and remediation plan for all private residences where children spend time as well as other places where they come together would cater for individual children who may not directly benefit from zonal remediation.

The broader implications of these findings can be considered in the context of a recent WHO [36] review of evidence to assist governments and other agencies to deal with the redevelopment of contaminated sites in urban settings. The report viewed the potential to remediate former industrial sites and contaminated land for urban revitalisation and development as a feasible alternative to using other limited land resources. The review concluded, inter alia, that there is a lack of practical advice about how to decontaminate a site. The remediation of contaminated lands to prevent environmental exposure requires a good understanding of the sources and pathways of exposure and elucidation of the specific pathways in a given context that might guide local action, as exemplified by Armijos et al. in Ecuador [37]. When children are affected, such as with lead, clarifying where they spend time in a community in addition to how they spend time may be an important piece of information that could assist preventive action in specific settings.

### Study Limitations

The data on the key study variables concerning the amount of time spent away from home each week were based on self-reporting rather than the use of prospective diary records. The potential for overestimation was mitigated by including an option to record a time range rather than a single estimate over the past month. We used the minimum time as a conservative estimate for our calculations. We did not collect information on the lead hazard and risk behaviour of children at private residences outside the family home and thus were not able to estimate the level of risk for individual children at these locations. The study did not include Australian Aboriginal and Torres Strait Islander children (19% of the at-risk population) who attended a second screening clinic run by the local Aboriginal Community Controlled Health Service [38]. 

## 5. Conclusions

This study provides important information about where young children spend time when away from the family home in an Australian mining town and shows how this may contribute to their lead risk. Locally, further investigation of the lead hazard and risk behaviour of children at private residences outside the family home would assist in estimating the level of risk for individual children and in developing practical guidelines for the assessment and remediation of the lead hazard for private residences in a future primary prevention strategy. In other at-risk settings, this approach to investigating the potential sources and pathways of lead and other heavy metal exposures for young children may have merit where zonal remediation to achieve comprehensive cleanup is not feasible or may not be warranted.

## Figures and Tables

**Table 1:** Summary of the key characteristics of the study subjects and the primary place they visited outside the family home, Broken Hill, 2018.

Factor		Age Group				Total
		Infants (*n* = 8)	1-Year-Olds (*n* = 30)	2-Year-Olds (*n* = 16)	3-Year-Olds (*n* = 11)	(*n* = 65)
Characteristics of the children						
Gender	Male Female	4 4	12 17	5 10	5 6	26 (40%) 37 (60%)
Blood lead level (µg/dL)	<5 ≥5	6 2	14 16	7 9	7 4	34 (52%) 31 (48%)
Family home						
Location lead hazard zone ^#^	High Lower	2 6	5 25	4 12	1 10	12 (18%) 53 (82%)
Primary place visited outside the family home *						
Type of place	Private residence Other	5 3	20 10	11 5	8 3	44 (68%) 21 (32%)
Reason for visit	Care Social Other	1 5 2	12 11 7	8 7 1	6 5 –	27 (42%) 28 (43%) 10 (15%)
Number of visits per week	<1 1–3 4 ^+^	1 6 1	8 12 10	6 3 7	1 4 6	16 (25%) 25 (38%) 24 (37%)
Time spent (h/week)	Median Range	7.5 1.5–16	13 1–84	13 1–84	15 1.5–45	14 1–84
Percentage of the total time outside home	Median Range	58 24–88	53.5 27–100	62.5 28–100	60 36–92	58 24–100

Note: ^+^ missing values: 2; ^#^ location of the family home based on the soil lead hazard zones—highest: zones 1, 2; lower: zones 3,4,5; * location where the child spent most time outside the family home in the previous month: private residence or other (childcare facility, family day care, recreational, commercial).

**Table 2:** Details of the places where young Broken Hill children spent time outside the family home, 2018.

	Number of Children		
	Primary Place *	Second Place	Third Place
Type of place			
Private residence	44 (68%)	25 (38%)	20 (31%)
Child care facility	10 (15%)	12 (18%)	–
Other	11 (17%)	25 (38%)	35 (54%)
Time per week (hours)			
Median	14	3	2
≥5 h	47 (74%)	19 (29%)	8 (13%)
≥10 h	36 (55%)	9 (14%)	1 (2%)
Duration of visits (months)			
Median	12	12	12
≥6 months	55 (86%)	51 (82%)	47 (85%)

Note: * primary place based on the time spent at a location.

**Table 3:** Relationship to the occupant of the most commonly visited private residence where a child spent time outside the family home, 2018.

Home Occupant	Time Spent at the Residence Per Week			Total
	<5 h	5–9 h	10+ h	
Grandparent	9 (56%)	9 (69%)	21 (75%)	39 (68%)
Other relative	1 (6%)	2 (15%)	5 (18%)	8 (14%)
Friend	6 (38%)	2 (15%)	2 (7%)	10 (18%)
Total	16 (100%)	13 (100%)	28 (100%)	57 (100%)

## Data Availability

Due to privacy reasons, the data are not available for sharing.
